# LPIAT, a *lyso*-Phosphatidylinositol Acyltransferase, Modulates Seed Germination in *Arabidopsis thaliana* through PIP Signalling Pathways and is Involved in Hyperosmotic Response

**DOI:** 10.3390/ijms21051654

**Published:** 2020-02-28

**Authors:** Denis Coulon, Lionel Faure, Magali Grison, Stéphanie Pascal, Valérie Wattelet-Boyer, Jonathan Clark, Marina Le Guedard, Eric Testet, Jean-Jacques Bessoule

**Affiliations:** 1 Laboratoire de Biogenèse Membranaire, UMR5200 Centre National de la Recherche Scientifique, Université de Bordeaux, 33883 Villenave d’Ornon, France; LFaure@twu.edu (L.F.); magali.grison@u-bordeaux.fr (M.G.); stephanie.pascal@u-bordeaux.fr (S.P.); valerie.wattelet@u-bordeaux.fr (V.W.-B.); marina.le-guedard@u-bordeaux.fr (M.L.G.); eric.testet@bordeaux-inp.fr (E.T.); jean-jacques.bessoule@u-bordeaux.fr (J.-J.B.); 2 Bordeaux INP, ENSTBB, 33076 Bordeaux, France; 3 Department of Biology, Texas Woman's University, Denton, TX 76204-5799, USA; 4 Babraham Institute, Babraham Research Campus, Babraham, Cambridge CB223AT, UK; jonathan.clark@babraham.ac.uk

**Keywords:** Acyltransferase, phosphoinositides, seed germination, hyperosmotic stress, ABA, aleurone-like cells

## Abstract

*Lyso*-lipid acyltransferases are enzymes involved in various processes such as lipid synthesis and remodelling. Here, we characterized the activity of an acyltransferase from *Arabidopsis thaliana* (LPIAT). In vitro, this protein, expressed in *Escherichia coli* membrane, displayed a 2-*lyso*-phosphatidylinositol acyltransferase activity with a specificity towards saturated long chain acyl CoAs (C16:0- and C18:0-CoAs), allowing the remodelling of phosphatidylinositol. *In planta*, *LPIAT* gene was expressed in mature seeds and very transiently during seed imbibition, mostly in aleurone-like layer cells. Whereas the disruption of this gene did not alter the lipid composition of seed, its overexpression in leaves promoted a strong increase in the phosphatidylinositol phosphates (PIP) level without affecting the PIP2 content. The spatial and temporal narrow expression of this gene as well as the modification of PIP metabolism led us to investigate its role in the control of seed germination. Seeds from the *lpiat* mutant germinated faster and were less sensitive to abscisic acid (ABA) than wild-type or overexpressing lines. We also showed that the protective effect of ABA on young seedlings against dryness was reduced for *lpiat* line. In addition, germination of *lpiat* mutant seeds was more sensitive to hyperosmotic stress. All these results suggest a link between phosphoinositides and ABA signalling in the control of seed germination

## 1. Introduction

In eukaryotic cells, phosphoinositides (Pins) are a minor phospholipid group (less than 1% of total membrane lipids) synthesized after several sequential phosphorylation/dephosphorylation from phosphatidylinositol (PI) by various kinases/phosphatases. In plants, different Pins have been identified: three phosphatidylinositol phosphates (PIP) (PI3P, PI4P, PI5P) and two phosphatidylinositol bisphosphates (PIP_2_), (PI(3,5)P_2_, PI(4,5)P_2_) (for review [1,2]). These lipids are involved in numerous cellular processes such as cell polarity [3], vacuolar trafficking [4], light-induced stomatal opening [5] and root hair tip growth [6]. They are also described as key regulatory molecules in plant-responses during abiotic stresses such as wounding [7], heat stress [8] and hyperosmotic stress [9,10]. For further insights on cellular functions of Pins in plants, the reader is referred to the following reviews [1,2,11,12,13].

Plant stress responses promote transient modifications in PI / Pins levels and also in their fatty acid composition. For instance, heat shock stress induced an increase of PIP_2_ content in tobacco cell culture (BY-2) by a factor 4 after only 30 minutes at 40 °C [8]. In *Arabidopsis*, the level of PI4P and PI(4,5)P_2_ increased by 2-3 fold, four hours after wounding [7], while salt treatment (e.g., NaCl) on cells of different plant species was associated with an increase in the PIP_2_ level [14,15,16,17]. The unsaturation of fatty acid moieties within the Pins increased after wounding or hyperosmotic stress [7,9,18].

The fatty acid composition of PI, precursor of Pins, is remarkably different from that of other phospholipids (i.e., phosphatidylcholine, phosphatidylglycerol, etc.). In fact, in animals, plants and yeast (e.g., *Saccharomyces cerevisiae*), PI has a high content of saturated fatty acids [19,20,21]. In plants, the most abundant fatty acid within PI is palmitic acid which represents up to 40% of the total fatty acid of this phospholipid [20]. This unusual composition of PI is thought to result from sequential deacylation-reacylation reactions mediated by *lyso*-phosphatidylinositol acyltransferase (LPIAT). The genes involved in this remodelling have been identified in *Saccharomyces cerevisiae* [21] and *Caenorhabditis elegans* [22]. Several examples highlighted the implication of this deacylation-reacylation process in different cellular functions. For instance, an alteration in the PI remodelling in stem cell-like epithelial cells impaired asymmetric cell division and therefore proper intracellular retrograde transport [22]. Mice deficient for *LYCAT* gene, closest homolog of the *sn1*-*lyso-*PI acyltransferase from *Caenorhabditis elegans*, or mice knock-out for the *LPIAT1* gene have an altered fatty acid composition of one or more of their PI, PIP and PIP_2_ lipids [23,24]. The effects of the fatty acid composition (saturated vs. unsaturated) at the *sn1* position of the PI group in the *lycat* KO mice remain unknown since no phenotype has been observed for those animals. Interestingly, when the *LPIAT1* gene is disrupted in mice, the arachidonoyl content at the *sn2* position of PI and PIP2 is strongly diminished in liver and brain, but not for PIP species. Unlike *lycat* KO mice, alteration of the acyl composition in *lpiat1* KO mice is accompanied by severe phenotypes such as developmental brain defects. This emphasized the importance of the acyl composition at the *sn2* position of PI and PIP_2_ for brain functions in mice [24].

In *planta*, different *lyso-*lipid acyltransferases (*lyso*-lipid AT) have already been identified such as a *lyso-*phosphatidylcholine AT [25,26], *lyso*-phosphatidic acid AT [27,28,29], *lyso*-phosphatidylethanolamine AT [30] and an acyltransferase with a broad specificity of *lyso*-lipid as acyl acceptor [31]. However, to our knowledge, no *lyso-*phosphatidylinositol AT has been characterized in plants.

The present work shows that the A*rabidopsis thaliana* acyltransferase LPIAT (At3g05510) catalyses PI remodelling with a strong specificity towards saturated acyl-CoAs when expressed in *E. coli* membranes. *In planta*, *LPIAT* is expressed in seed during imbibition and more precisely mostly in the endosperm, a peripheral aleurone-like cell layer surrounding the embryo. The overexpression of LPIAT leads to an accumulation of PIP species in leaves, suggesting the involvement of this protein in signalling processes. Interestingly, germination study of *LPIAT* mutants showed the implication of this protein in the initiation of seed germination and in ABA and hyperosmotic stress responses. 

## 2. Results and Discussion

### 2.1. Identification of LPIAT in Arabidopsis thaliana

By sequence homology with the *lyso*-phosphatidylcholine acyltransferase from *S. cerevisiae* (YPR140wp), characterized in our laboratory [32], we found different candidate genes potentially involved in acyltransferase activity. Among those candidates, the protein coded by *At3g05510* sequence (*LPIAT*) contained 3 of the 4 conserved domains generally associated with glycerolipid acyltransferase activity [33]. The structure of this gene is shown in Figure S1. Interestingly, we observed the presence of block I (NHX4D) and III (FPEGT) described as binding sites for *lyso*-lipids and acyl-CoAs, respectively [33,34]. The cDNA of *LPIAT* encodes for a predicted protein of 448 amino acids with a molecular mass estimated to 50.3 kDa with an isoelectric point calculated at 9.95, with no evident targeting signal. Based on the results of 18 different programs, no real transmembrane sequences have been predicted (aramemnon.org). According to the consensus data, only one TM sequence with a low score (0.24) has been predicted between the 87 to 107 amino acids. No myristoylation, prenylation, GPI anchor sites or post-translational modifications have been predicted using different programs such as NMT (mendel.imp.ac.at/myristate/SUPLpredictor.htm), Myristoylator (http://web.expasy.org/myristoylator/) or PrePS (mendel.imp.ac.at/sat/PrePS/index.html), PredGPI (http://gpcr.biocomp.unibo.it/predgpi/pred.htm) and GPI Som (http://gpi.unibe.ch/).

Transient expression in epidermal cells of tobacco leaves (*Nicotiana tabacum)* of LPIAT tagged at either N-ter or C-ter position with the YFP protein showed co-localization with the plasma membrane H^+^-ATPase (PMA4) fused with GFP protein. These results suggested a plasmalemma localization of LPIAT (Figure S2)

### 2.2. LPIAT is a sn-2 lyso-Phosphatidylinositol Acyltransferase with High Specificity for Saturated acyl-CoAs 

**Figure 1:**
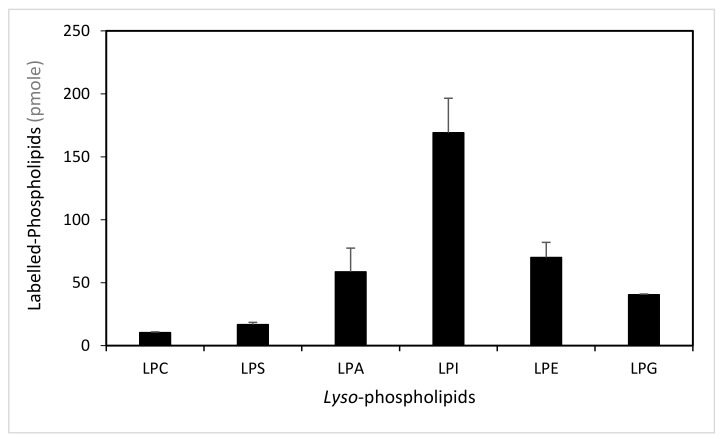
Determination of the *lyso*-phospholipid specificity. Labelled-phospholipids were synthesized by in vitro acylation of *lyso*-phospholipids, catalysed with membranes from *E. coli* transformed by pET-15b::*LPIAT* plasmid (100 µg protein/assay) corrected by the amount of labelled phospholipids obtained with membranes transformed by empty pET15b. Assays were carried out with 3 nmol of [^14^C]-C18:1-CoA and 2 nmol of *lyso*-phospholipids. After 60min incubation, lipids were extracted and analysed by thin-layer chromatography (TLC) followed by radioimaging. Values represent mean ± SD of three biological replicates. LPC, *lyso*-phosphatidylcholine; LPS, *lyso*-phosphatidylserine; LPA, *lyso*-phosphatidic acid; LPI, *lyso*-phosphatidylinositol; LPE, *lyso*-phosphatidylethanolamine; LPG, *lyso*-phosphatidylglycerol.

To determine the ability of LPIAT to synthesize phospholipids from *lyso*-phospholipids, we performed enzymatic assays using *E. coli* membrane preparation enriched with LPIAT (Figure S3) in presence of labelled acyl-coenzyme A as acyl donor ([^14^C]-C18:1-CoA) and different unlabelled *lyso*-phospholipids as acyl acceptors (Figure 1). All *lyso*-phospholipids are acylated in presence of [^14^C]-C18:1-CoAs, however LPIAT displays a strong specificity for the *lyso*-phosphatidylinositol compared to the others *lyso*-phospholipids tested (from 2.4 to 10-fold higher).

Moreover, we investigated the specificity of this protein for acyl-CoAs with different carbon chain length and degree of unsaturation. Since not all radiolabelled [1-^14^C]-acyl-CoAs are available, we used an isotopic dilution strategy to compare the activity of LPIAT in presence of both radiolabelled [1-^14^C]*-*C18:1:CoA and unlabelled acyl-CoA [32].

**Figure 2:**
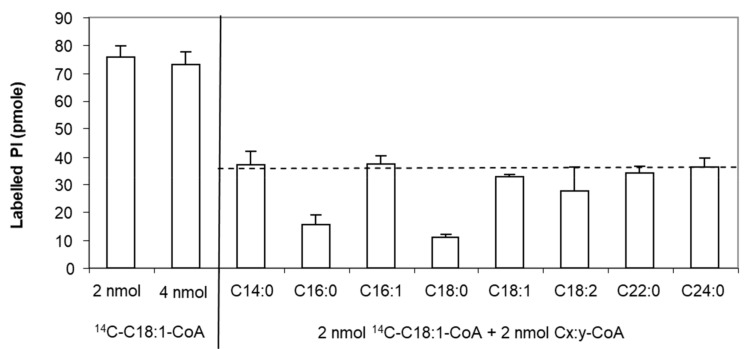
Determination of the acyl-CoA specificity. In vitro acylation of *lyso*-phosphatidylinositol was catalysed with membranes from *E. coli* transformed by pET-15b::*LPIAT* plasmids (10 µg protein/assay) corrected by the amount of labelled phospholipids obtained with membranes transformed by empty pET15b. Assays were carried out with 2 nmol of [^14^C]-C18:1-CoA, 2 nmol unlabelled acyl-CoA and 4 nmol of LPI. After 10min incubation, lipids were extracted and analysed by TLC using chloroform/methanol/1-propanol/methyl acetate/0.25% aqueous KCl (10:4:10:10:3.6, v/v) as solvent followed by radioimaging. Values represent mean ± SD of 3 biological replicates with two technical replicates. The dashed line represents the theoretical level of labelled PI reached in presence of 2 nmol [^14^C]-C18:1-CoA and 2 nmol C18:1-CoA.

We determined the experimental conditions (i.e., C18:1-CoA concentration, incubation time and protein concentration) allowing the saturation of the enzyme and leading to less than 10% conversion of substrates. We found that 4 nmoles of LPI and 2 nmoles of acyl-CoA led to a saturated state of the protein and that 4 nmoles acyl-CoA did not inhibit the enzyme by an excess of substrate. Using 2 nmoles of labelled C18:1-CoA and 2 nmoles of unlabelled C18:1-CoA, a 2-fold decrease in the labelled PI synthesized is observed consequently to the isotopic dilution of the acyl donor (Figure 2). For other unlabelled acyl-CoAs, a higher decrease in the amount of labelled PI than observed with the unlabelled C18:1-CoA means that LPIAT displays a higher specificity towards these unlabelled acyl CoAs than towards C18:1-CoA. We observed a strong reduction of the amount of labelled PI synthesized in presence of C16:0-CoA and C18:0-CoA (Figure 2), highlighting a higher specificity of LPIAT towards these substrates. On the contrary, the use of other saturated acyl-CoAs (with medium or very long chain length) or unsaturated acyl-CoAs did not alter the incorporation of C18:1-CoA into PI.

**Figure 3:**
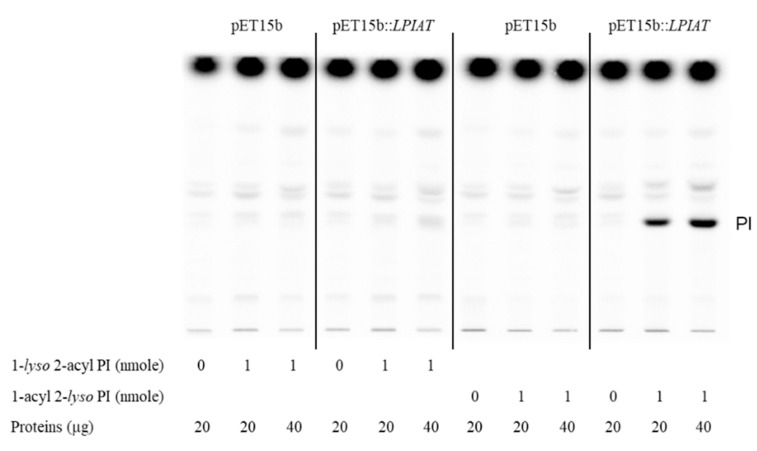
Determination of the regiospecificity of acyltransferase activity catalysed by LPIAT on *lyso*-phosphatidylinositol. Microsomal membrane proteins (20 or 40 µg) from *E. coli* transformed by empty pET15b or pET-15b::*LPIAT* plasmids were incubated with [^14^C]-palmitoyl-CoA (1 nmol) in the absence or in the presence of 1-*lyso* 2-acyl PI or 2-*lyso* 1-acyl PI (1 nmol). After 10 min of incubation, lipids were extracted and analysed by TLC followed by radioimaging. Results are from one experiment representative of three experiments performed with independent microsome preparations.

Moreover, using different acyl acceptors (1-*lyso* 2-acyl phosphatidylinositol and 2-*lyso* 1-acyl phosphatidylinositol), we showed that LPIAT expressed in *E. coli* membranes acylated preferentially 1-acyl 2-*lyso* phosphatidylinositol indicating a high specificity for the *sn2* position (Figure 3).

To date, several *lyso*-phosphatidylinositol acyltransferases have been characterized. In animal tissues, a 2-lyso-phosphatidylinositol acyltransferase, displaying a marked preference for unsaturated-CoA, is ubiquitously expressed [35,36]. Disruption of this gene promotes a decrease in PI and phosphoinositide species containing arachidonic acid (C20:4) and leads to abnormal brain morphology [37]. A 1-*lyso* phosphatidylinositol acyltransferase has also been previously described in animal cells and appeared to be crucial for asymmetric divisions [22]. *Saccharomyces cerevisiae* psi1Δ strain, mutated for a *lyso*-phosphatidylinositol acyltransferase activity, showed alteration in the proportion of some molecular species of PI and phosphoinositides with stearic acyl chains incorporated at the *sn*-1 position of PI. This induces disturbances of intracellular trafficking, alteration of budding pattern and defects of actin cytoskeleton organization [38].

In leaves, phosphatidylinositol fatty acid composition is characterized by a high content of palmitic acid at *sn-*1 position while linoleic and linolenic acids are mostly abundant at *sn-*2 position [39]. Until now, no LPIAT has been identified, whatever the *sn*-specificity. The fatty acid composition of PI and phosphoinositides depends on the specificity of two phosphatidylinositol synthase isoforms, PIS1 and PIS2, for saturated or monounsaturated and polyunsaturated CDP-DAG substrates, respectively [40]. The activity of LPIAT, which incorporates a saturated fatty acid at *sn2* position, may play a role in a very specific pathway or tissue and may modify PI fatty acid composition during a defined process.

### 2.3. LPIAT is Involved in Phosphoinositide Metabolism

According to publicly available expression data, *LPIAT* was mostly expressed in seeds (Genevestigator (www.genvestigator.com); data hosted at www.arabidopsis.org). We measured the transcript level of *LPIAT* in seeds and leaves by semi-quantitative RT-PCR (Figure S4). 

The expression of *LPIAT* in dry seed was 55-fold higher compared to leaves from one-week-old plants and transcript level in leaves decreased over time. Interestingly, we observed a transient increase of mRNA transcript level during imbibition with a maximal expression level after one hour of imbibition. Our results are consistent with the data of gene expression reported on BAR (http:bar.utoronto.ca/eplant/).

**Figure 4:**
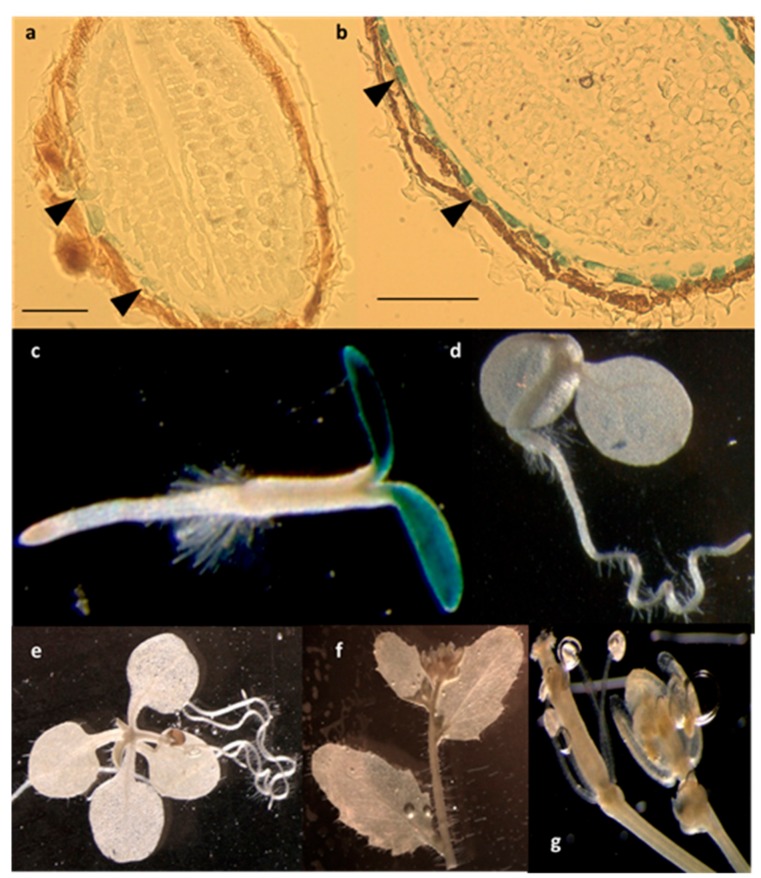
Spatial expression patterns of *LPIAT* in transgenic *Arabidopsis* harbouring the *LPIAT* promoter fused to the β-glucuronidase (GUS) gene. Promoter activity was visualized by histochemical GUS staining on (**a**) dry seed, (**b**) imbibed seed, (**c**) 2-day-old seedling, (**d**) 4-day-old seedling, (**e**) 9-day-old seedling, (**f**) cauline leaves and inflorescence, (**g**) flower. Scale bar: 100 µm. Arrow heads in (a) and (b) show cells from the aleurone-like layer.

Using a β-glucuronidase (GUS) reporter approach, we observed the expression pattern of the *LPIAT* gene by generating transgenic *Arabidopsis* lines expressing the β-glucuronidase (GUS) reporter gene under the control of the *LPIAT* promoter. As observed in Figure 4, *LPIAT* promoter was more active in imbibed seed than in dry seed, and mainly in the endosperm layer surrounding the embryo (Figure 4a,b). In 2-day-old seedlings, expression occurred only in cotyledons (Figure 4c). No glucuronidase activity was detected in the shoot and the root apical meristems, nor in the primary root or hypocotyl (Figure 4c). At more advanced developmental stages, *LPIAT* was anymore expressed whatever the tissue (Figure 4d–g). It would be very informative to analyse the expression pattern of *LPIAT* during the post-germination time to determine when *LPIAT* begins to be expressed in the cotyledons. This could be achieved by performing combined GUS and RT-PCT experiments.

As imbibition is the first step of seed germination [41] and as endosperm tissue plays an active role in the regulation of seed germination [42], our results suggest that LPIAT is involved in seed germination. In agreement, the upstream sequence of *LPIAT* shares a putative G-box (CACGTG), binding site of transcription factors (AtbZIP44 and AtPIL5) that regulate in the endosperm the expression of genes involved in the germination [42].

As LPIAT has an acyltransferase activity in vitro, we compared the lipidome of seeds before and after imbibition from various *LPIAT* mutants (i.e., TDNA line (*lpiat),* overexpressors in the wild-type background (LPIATOx) or in the KO background (*lpiat*:LPIATOx, rescue line)), focusing our attention on PI level/fatty acid composition (Figure S5). Consistent with the literature [43], in wild-type seeds, PI represented 22% of total phospholipids and was mainly esterified with C16:0 and C18:2 (34% and 37%, respectively).

We did not observe any significant difference in the seed lipidome, and in the fatty acid composition of PI in particular, whatever the expression level of *LPIAT* and the hydration state of the seed. The absence of a lipid phenotype can be explained by the very transient and located expression of *LPIAT*. Indeed, as *LPIAT* expression occurs mainly in the aleurone-like cells, only few cells in the seed may have altered lipid profile and it may be hidden by the unaltered lipid pool from other tissues in the seed.

In addition, in overexpressing lines, the absence of significant lipid alterations may be also attributed to the relatively low activity of the 35S promotor in seed (the expression level is only 3 and 1.7 fold higher compared to wild type for *lpiat*:LPIATOx and LPIATOx, respectively) (Figure S6).

**Figure 5:**
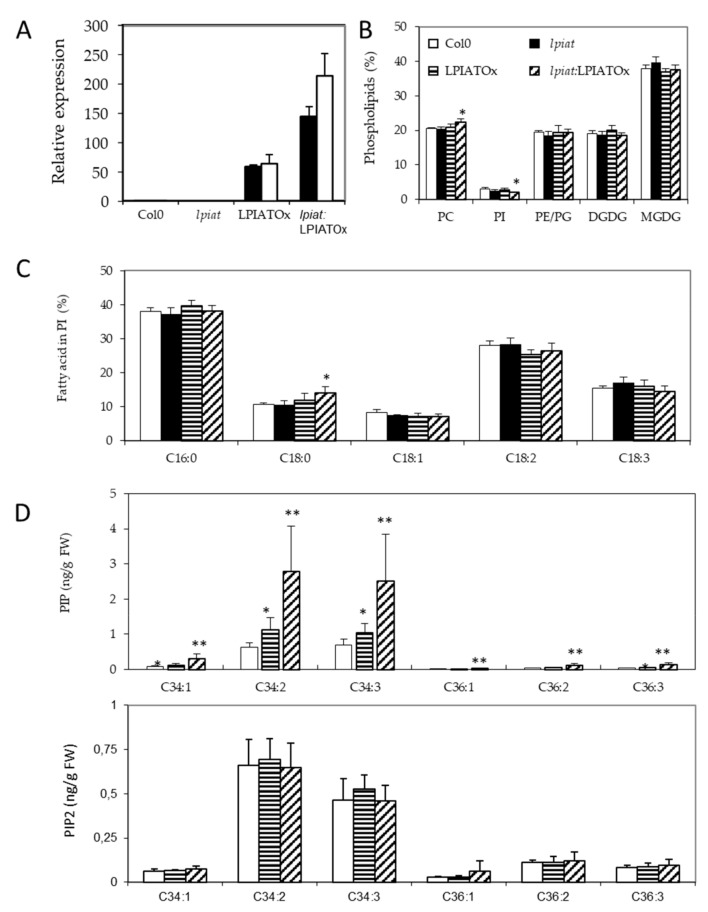
Impact of LPIAT on lipid metabolism. (**A**), Relative quantification of *LPIAT* expression in wt, *lpiat*, LPIATOx (overexpression in wild-type background) and *lpiat*:LPIATOx (overexpression in knock-out background) in leaves from 1 week-old plants, normalized to two reference genes (actin and eIF4A-1). Relative expression quantities are represented related to wild type level at 1 week, which was set to one. Values represent mean ± SD of three technical replicates for two biological replicates (black and white boxes). (**B**), Phospholipid analysis of wt, *lpiat* or overexpressing leaves. Lipids from leaves from 3 week-old plants were quantified by GC-FID after transesterification. Values represent mean ± SD for four biological replicates. (**C**), Phosphatidylinositol (PI) fatty acid composition (in % of total FA). Values represent mean ± SD for four biological replicates. (**D**), Effect of *LPIAT* overexpression on the molecular fatty acid composition of PIP (top panel) and PIP2 (down panel) in leaves. Phosphoinositides were extracted from leaves from 3-week-old plants by acidic extraction and analysed by LC MS/MS after derivatization. Values represent mean ± SD for five biological replicates. Statistically significant differences from the wild type are indicated by * *p* < 0.05 or ** *p* < 0.01 as determined by Wilcoxon’s-test.

As the 35S-promotor is highly efficient in leaves (Figure 5A), we sought for a lipid phenotype in overexpressing leaves. The phospholipid and galactolipid contents are weakly affected by modification in *LPIAT* expression level, excepted a slight but significant decrease of PI (Figure 5B) in *lpiat*:LPIATOx line which has the highest expression level. A significant increase in the C18:0 content of PI is also observed (Figure 5C). On the other hand, the determination of the phosphoinositide composition revealed an increase in phosphatidylinositol monophosphate (PIP) content associated with the expression level of *LPIAT* (Figure 5D). 

On the contrary, the phosphatidylinositol bisphosphate (PIP2) content remained unchanged whatever the lines. C34:2 and C34:3 species are the major phosphoinositide detected in both PIP and PIP2 pools (in PIP, 43.0% and 46.5%; in PIP2, 46.7% and 32.5%, respectively). C16:1 fatty acid is almost only associated with plastidial phosphatidylglycerol in plants, C16:2 is quasi absent in leaf tissues, C16:3 is only associated with MGDG, and because C18:2 and C18:3 are the major fatty acids [43], our results greatly suggest that PIP species that increased in overexpressing lines are very likely esterified with C16:0 fatty acid moiety and a C18:2 or C18:3 on the glycerol backbone (for the C34:2 and C34:3 species, respectively). Noteworthy, the amount of all PIP species increased in the same extent in the overexpressing lines.

These results suggest that LPIAT leads to a remodelling of PI, which becomes more saturated. These saturated species (and mainly C16:0-containing species) could be preferentially phosphorylated by a PI kinase, promoting an increase of defined PIP species in the endosperm cells from seed.

As the aleurone-like cell layer is known to be involved in the control of seed dormancy and germination, especially by synthesizing and releasing ABA towards the embryo, that blocks the germination [44,45], we studied seed germination in function of the expression level of *LPIAT*. Moreover, since phosphoinositides mediated plant-response against stress [46], the development of young seedlings throughout drought and osmotic stresses was also investigated.

### 2.4. LPIAT Delays Seed Germination and Reduces Root and Hypocotyl Growth

**Figure 6:**
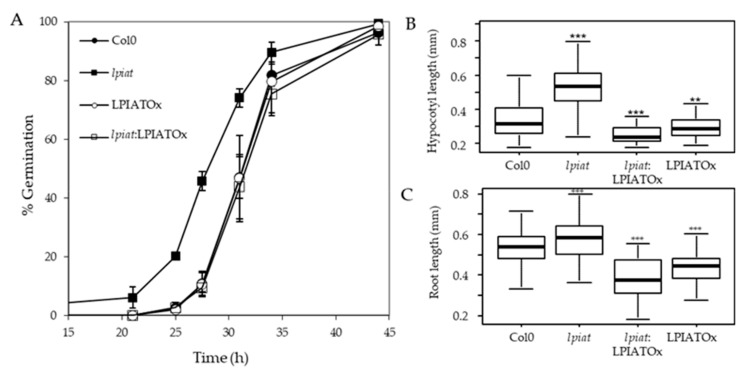
Germination and growth phenotype. (**A**), Seed germination in Col0, *lpiat,* LPIATOx and *lpiat:*LPIATOx lines. After 3 days stratification, seeds were germinated on 1/2 MS medium, 0.8% agar and 1% Sucrose. About 100 seeds were sowed by plates. % germination was estimated by plates as judged by radical extrusion. Values are means ± SD (*n* = 3). (**B**), Hypocotyl length from 3-day-old dark-grown seedlings from wild-type and the indicated *LPIAT* mutant lines. Seeds were germinated and grown in the dark for 3 days on 1/2 MS medium, 0.8% agar and 1% Sucrose. (**C**), Primary roots were measured after 3 days. Values are means ± SD (*n* = 60) from one of three independent experiments that gave similar results. Wilcoxon’s test comparisons between altered-*LPIAT* expression level mutants and the wild type were performed. ***, *p*-value was < 0.005; **, *p*-value was < 0.01.

We examined the germination rate of seeds that had been harvested from different plants grown during the same period. Seeds were plated on ½ Murashige and Skoog medium, stratified for 3 days at 4 °C in the dark and then grown in the light. Germination evaluated by radical extrusion was followed for 48h (Figure 6A). All the seeds achieved 100% germination within 48 h but *lpiat* mutant germinated before the wild-type. In fact, after 29 h, the germination rate is 2.5-fold higher in *lpiat* mutant than in the wild-type whereas no difference was observed between LPIATOx line and wild-type. Noteworthy, the overexpression of LPIAT in the KO background (rescue line) restored the germination profile to that of the wild-type. 

To address whether this phenotype is associated with a modification of the seed coat permeability, we used a tetrazolium salt permeability assay [47]. Tetrazolium salt is a cationic dye naturally blocked by the seed coat. If entering in contact with the embryo, it is reduced forming a red product (formazan). After few minutes of incubation between seeds and the tetrazolium salt, we did not observe any significant increase of formazan production in the embryo of the *lpiat* seeds (Figure S7), demonstrating that the early germination of *lpiat* seeds was not associated with an increase of seed coat permeability.

We next examined the effect of *LPIAT* expression level on the hypocotyl length. After 3 day-growth in the dark, the hypocotyl length of *lpiat* line is longer than for the wild-type. This could be due to an early germination of seeds, but it can be noted that whereas LPIATOx, *lpiat*:LPIATOx and wild-type lines displayed similar germination rate, the hypocotyl length of the overexpressing and rescue lines are shorter than in wild-type (Figure 6B). This opposite phenotype between *lpiat* mutant and the overexpressing and rescue mutants clearly demonstrated that these results were caused by an alteration of the expression level of *LPIAT*. However, after 7 days of growth, the length of the hypocotyls between the different lines were similar (data not shown). We also measured a significant difference of *lpiat*, LPIATOx, *lpiat*:LPIATOx primary root length of young seedlings compared to wild-type (Figure 6C). As for the hypocotyl length, this difference disappeared after 7 days of growth (data not shown).

Similar phenotypes have already been observed during germination of *Arabidopsis* seed mutated in myoinositol polyphosphate 5-phosphatase genes (5PTase1 and 5PTase2) [48]. These phosphatases hydrolyze 5-phosphates from a variety of myoinositol phosphate and phosphoinositide phosphate (PIP) substrates. Modifications of hypocotyl and primary root lengths are linked to a modification in the phosphoinositide pools (increase in Ins(1,4,5)P3 and slight decrease of PI, PI(4)P and PI (4,5)P2) [48]. Another study had also shown the involvement of phosphoinositides during rice seed germination [49]. The inhibition of PI3 kinase by wortmannin promotes a delay of rice seed germination. These studies reinforce the hypothesis that phosphoinositides and germination are closely related, and that LPIAT is involved in phosphoinositide metabolism through an unknown mechanism. Its activity may generate specific PI species that would be preferentially phosphorylated by different kinases or would allow the recruitment of proteins to the plasma membrane during germination. It would be very interesting to measure Pins and Inositolphosphate levels in isolated endosperm layer of seeds presenting different expression level of *LPIAT* to go further in the comprehension of these phenomenons.

### 2.5. Response of LPIAT Mutants to ABA

**Figure 7:**
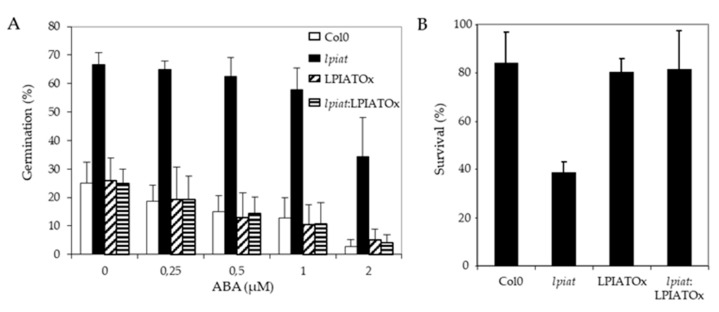
ABA-related modifications in seed germination and drought stress resistance in *LPIAT* mutants. (**A**), Germination rate from wild-type and the indicated *LPIAT* mutant seeds. Seeds were germinated for 29 h on 1/2 MS medium, 0.8% agar and 1% Sucrose. The medium was supplemented with various ABA concentrations. Values are means ± SD of 4 independent experiments. (**B**), Effect of a drought stress on survival rate of the different LPIAT mutants grown on a medium supplemented with ABA before the stress. Seeds were sown on filter laying on ½ MS medium, 0.8% agar and 1% Sucrose. After 3 days stratification at 4 °C, filters were transferred on the same medium supplemented or not by 5 µM ABA for 10 days. Filters were removed from medium and dried at room temperature for 6 h. Then, they were deposited on ½ MS medium, 0.8% agar and 1% Sucrose; Survivals were counted after 4 days. All the seedlings that were not initially transferred on medium supplemented by 5µM ABA died after the drought stress (not shown). Values are means ± SD of 5 independent experiments.

One possibility to explain the early germination of *lpiat* seeds is a lower sensitivity to ABA. To assess this hypothesis, seeds from wild-type, KO, overexpressing and rescue lines were plated on ½ MS medium supplemented with 0, 0.25, 0.5, 1 and 2 µM ABA and the germination score was measured after 29h, time corresponding to a high difference in germination rate observed previously (Figure 6A). As hypothesized, *lpiat* seeds are less sensitive to exogenous ABA. As shown in Figure 7A, on the medium containing 2 µM ABA, *lpiat* seeds have higher germination rate compared to wild-type seeds (34% versus 3% with supplemented ABA). In addition, in comparison with control conditions (without ABA supplementation), 2 µM ABA decreases the germination rate by 90% in wild-type but only by 44% in *lpiat* seeds. Noteworthy, the rescue line showed the same response to ABA treatment than the wild-type or the overexpressing line.

To go further, it would be interesting to determine if and how ABA regulates the expression of *LPIAT* during seed germination, and more precisely during imbibition. A positive control of *LPIAT* expression by ABA can explain the early germination observed with *lpiat* mutant. In agreement, micro-array data available on Genvestigator showed a high expression of *LPIAT* during seed imbibition in presence of ABA.

As ABA protects young seedlings against drought by blocking vegetative growth during early development [50], we examined the response of *lpiat* seedlings after a 6 h drought stress on 10 day-old seedlings grown in presence or absence of ABA. All seedlings that initially germinated and grew on medium without ABA died after the drought-stress, whatever the expression level of *LPIAT* (data not shown). On the contrary, if the seeds germinated and grew on a medium containing 5 µM ABA, more than 80% of wild-type, overexpressing and rescue seedlings survived to the drought stress, whereas the survival rate of *lpiat* seedlings is less than 40% (Figure 7B). This result confirmed than *lpiat* mutant is less sensitive to ABA than the other lines studied. 

Crosstalks between phosphoinositides and ABA signalling pathways have already been described in guard cells during stomatal movements [5,51], or during plant drought response [10] but few evidences have been mentioned during seed germination. In rice, the inhibition of PIP2-specific phospholipase C reduced the germination rate and seedling growth and, the addition of ABA prevented the gibberellin-induced formation of IP3 in isolated aleurone layer [52].

### 2.6. lpiat Seeds are Hypersensitive to Osmotic Stress

**Figure 8:**
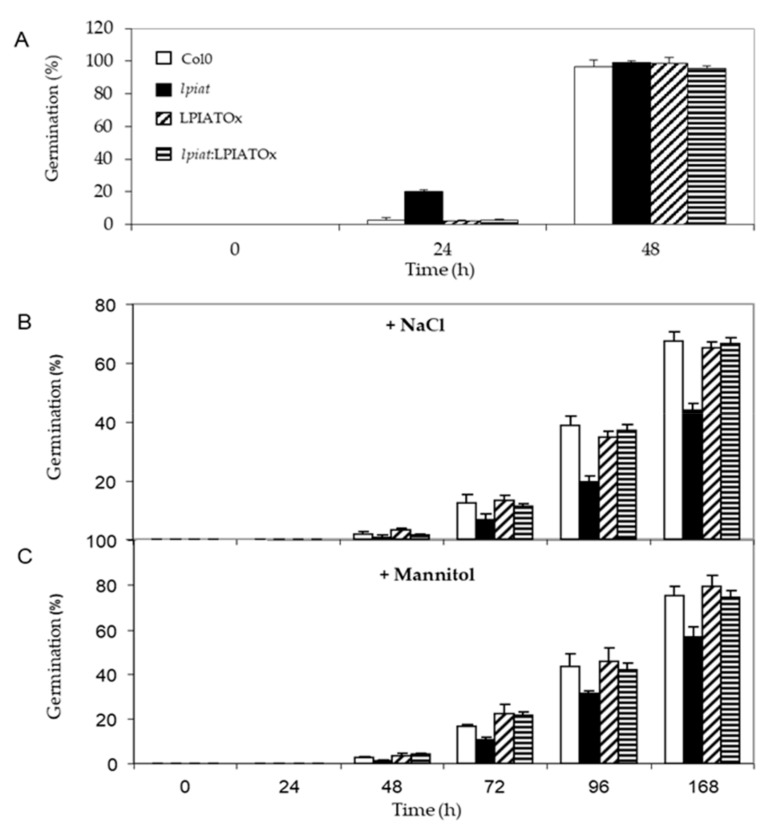
Germination efficiency of *LPIAT* mutants under osmotic/salinity stress. (**A**) Germination rate of wild type, *lpiat*, LPIATOx and *lpiat*:LPIATOx lines on MS media. (**B**) Germination rate on MS media containing 0.2 M NaCl. (**C**) Germination rate on MS media containing 0.4 M mannitol. Values are means ± SD of three independent experiments.

Seeds were sown on media containing either 0.2M NaCl or 0.4M Mannitol, leading to the same osmotic pressure in the medium. Germination rate was strongly reduced when plants grew on hyperosmotic media. After 48 h, about 100% germination was recorded under unstressed-growth conditions for all lines (Figure 8A), whereas, less than 10% occurred in presence of NaCl (Figure 8B). *lpiat* seeds were more sensitive to osmotic stress than wild-type seeds since their germination rate was reduced in a range from 35% to 50% (Figure 8B). This phenotype is truly due to the absence of LPIAT in the *lpiat* mutant because *lpiat*:LPIATOx displayed the same germination rate than the wild-type. (Figure 8B). A slightly less pronounced effect was observed with 0.4M Mannitol, showing that an ionic effect cumulated with the osmotic effect with 0.2 M NaCl (Figure 8C).

The roles of phosphatidylinositol and phosphoinositides in the plant response during osmotic stress have already been described but mostly during seedling growth. Thus, a 0.4 M NaCl treatment on *Arabidopsis* leaves promotes a transient decrease in unsaturated PI associated with a 7-fold and 2.4-fold enrichment in PI4P and PI4,5P2 respectively. This osmotic stress led to more than 66% of polyunsaturated fatty acids (C18:2 and C18:3) in PI4P species, which are fatty acids close to absent in PI4P from control plants [9,18]. The relationship between seed germination and phosphoinositide metabolism remains scarcely understood. Overexpression of a wheat PI4K (TaPI4KIIγ) in *Arabidopsis* enhanced drought and salt stress tolerance during seed germination and seedling growth [10]. On the contrary, when the orthologue gene (udbkγ7) is disrupted in *Arabidopsis*, the sensitivity to salt and ABA increased [10]. These results have also been observed when another PI4Kinase was unexpressed [46]. These phenotypes are in agreement with this study if we assumed that the PI species remodelled through LPIAT are preferentially phosphorylated by a PI kinase during seed imbibition. However, the amounts of the different Pins have not been determined in the different TaPI4KIIγ expression mutants and the author have not evidenced any lipid kinase activity. However, it should be noted that TaPI4KIIγ and *LPIAT* mutants displayed an antagonist response towards ABA signalling. In *lpiat* mutant, germination rate is less affected than the wild-type by ABA whereas in *udbkg7* mutant, seed germination is more reduced than in the wild-type. This difference pointed out that several mechanisms may be involved in these signalling processes.

A similar increase in sensitivity to osmotic stress has also been observed with the rof1^-^ knock out mutant during seed germination. ROF1 is an immunophilin playing an important role in the osmotic/salt stress responses of germinating *Arabidopsis* seedlings. This response occurred through interactions with specific phosphoinositides, mainly PI3P and PI3,5P2. [53]. 

## 3. Conclusions

In summary, we characterized the activity of a putative acyltransferase in *Arabidopsis thaliana*. This protein, encoded by *At3g05510* gene (*LPIAT*), has a 2-*lyso*-phosphatidylinositol acyltransferase activity and presents a high specificity towards saturated long chain acyl CoAs (C16:0- and C18:0-CoAs) when it is heterologously expressed in *Escherichia coli*. *In planta*, *LPIAT* gene is expressed mostly in a very narrow window of time during seed imbibition, and at a very precise location, the aleurone-like layer cells. Even if we did not evidence a lipid phenotype in seed, the overexpression of LPIAT in leaves causes an increase of the PIP content associated with a minor decrease of PI. In the same time, the PIP2 content is not affected. Disruption of this gene imputes seed germination. The overall results suggest that LPIAT remodels PI aleurone-like cells in seed, promoting an increase of saturated species of PIP through an undefined PI kinase. These modifications alter the initiation of seed germination as well as the response of seeds to hyperosmotic stress and ABA. The molecular mechanisms involved in such regulations as well as the nature of the PIP species affected (phosphorylation position) remains to be elucidated.

## 4. Experimental Procedures

### 4.1. Plant Material and Growth Conditions

A TDNA insertion mutant of *At3g05510* (*LPIAT*) was identified from the SALK collection (SALK_067860). The location and orientation of the TDNA insert was confirmed by DNA sequencing of PCR products amplified with TDNA (LBa1:5’-TGGTTCACGTAGTGGGCCATCG-3’) and gene-specific primers (LP: 5’-TATCTGCGTCCAAAATCAACC-3’ and RP: 5’-CTTCTCCACCGGCTTTTTATC-3’). Transgenic *Arabidopsis* was generated by floral-dip method using *Agrobacterium tumefaciens* (strain C58C1) transformed for the different constructs used for this study [54]. *Arabidopsis* seeds were grown on MS medium supplemented with 0.7% agar, 2.5 mM MES-KOH, where required—NaCl or mannitol, pH 5.7.

Seeds used for phenotypic assays were harvested from plants grown in parallel in a growth room and ripened for 6 weeks at room temperature. Seeds were surface-sterilized and plated on 1/2 Murashige and Skoog salt solution (pH 5.7) containing 0.7% agar, 2.5 mM MES-KOH and 1% sucrose. Seeds were stratified on plates at 4 °C for 3 days and germinated at 23 °C in the light or dark. Radicle protrusion through the seed coat was used as the criterion for the assessment of differences in germination between wild type and *LPIAT* mutant seeds. Seedlings were removed from plates at the indicated times and hypocotyl measurements were made at the indicated times with photographs and Image J software. For primary root length measurements, after 3-day stratification, plates were grown vertically in the dark or light. After 3-day growth, primary roots were measured. For ABA sensitivity experiments, ½ MS medium was supplemented with an ethanolic ABA solution at a final concentration of 0.25, 0.5, 1 or 2 µM ABA. Seedlings resistance to hydric stress was performed as described by [50]. Briefly, seeds were sown on absorbent papers laid on MS media. After 3 days at 4 °C, the absorbent paper was transferred on media supplemented or not with 5 µM ABA for 10 days. Plants were then water-stressed for 6 h by laying absorbent papers in a sterile fume-hood before being allowed to recover in normal medium for 4 more days.

### 4.2. Molecular Biology Constructs

The *LPIAT* sequence was amplified using the Phusion^TM^ High-Fidelity DNA polymerase using a set of sense and antisense primers containing the appropriate restriction sites to clone the sequence of interest in the pET-15b vector (Novagen, Merck Biosciences, Badsoden, Germany). The PCR product was purified with ultra Clean^TM^ GelSpin^TM^ DNA extraction kit (MO BIO laboratories, Carlsbad, CA, USA) according to the manufacturer’s protocol. This product of purification and pET-15b vector were digested using endonuclease enzymes Nco1 and Xho1 (from Biolabs), and the 5’ extremities were dephosphorylated (phosphatase alkaline, Biolabs). Then the vector was purified using UltraClean^TM^ Standard Mini Plasmid Prep Kit^TM^ (MO BIO laboratories, Carlsbad, CA, USA) according to the manufacturer’s instructions. All constructs were verified by sequencing. Finally the insert was introduced into pET-15b vector by ligation (T4 DNA ligase of Biolabs). C41 (DE3) *E.coli* bacteria (Avidis, Saint-Beauzire, France) was further transformed with the pET-15b containing the *LPIAT* sequence by heat shock transformation. Independent clones were obtained under ampicillin selection.

To obtain the constructions needed for tissue and cellular locations, the cDNA of *LPIAT* or the promoter of this gene (565bp before the start codon) was amplified using the PhusionTM High-Fidelity DNA polymerase and a set of sense and antisense primers containing the attB region. Using Gateway technology, the PCR products were cloned in the pDONR221 vector. After sequencing, the pDONR221 containing the sequence of interest (cDNA or gene promoter) were purified and used for the LR reaction with various destination vectors: pK7WG2D (for p35S:*LPIAT*), pK7WG2 and pK7WGF2 (for p35S:GFP-*LPIAT* in Cter or Nter respectively) or pKGWFS7 (p*LPIAT*:GUS).

### 4.3. Bacteria Growth and Proteins Induction Conditions

C41 (DE3) *E. coli* bacteria expressing plasmid pET15b::*LPIAT* or empty pET15b were grown overnight in LB medium with 0.1 mg/mL ampicillin at 37 ***°***C, at 200 rpm. After 1/20 dilution, the culture was continued in 250 mL flasks at 37 °C. Induction of *LPIAT* was done with 1 mM IPTG when the culture reached an absorbance OD_600_ = 0.6. After 3 h at 30 °C, cells were harvested by centrifugation and the pellet was resuspended in 500 µL of water.

### 4.4. Membrane Purification and Acyltransferase Assays

Fifty mL of *E. coli* culture were centrifuged and the pellet was resuspended in 2.5 mL 0.2 M Tris-HCl (pH 8) buffer. Then, 2.5 mL of 0.2 M Tris-HCl (pH 8), 1 M sucrose, 1 mM EDTA buffer was added and gently mixed before the addition of 25 μL lysozyme (1 g/L) and completed with 10 mL of water. Digestion of cell walls was performed at room temperature under gentle shaking for 30 min. Membranes were spun down by centrifugation for 15 min at 150,000 *g*, using a Hitachi centrifuge. Pellet was washed with 50 mM Tris-HCl (pH 8) buffer and then centrifuged at 4 °C for 15 min at 150,000 *g* (using a Hitachi centrifuge) and resuspended in 50 mM Tris-HCl (pH 8) buffer. The protein concentration was determined using the Lowry method against a BSA standard curve.

The *lyso*-phospholipid acyltransferase activity was measured for 60 min at 30 °C in 100 μL of 50 mM Tris-HCl buffer (pH 8) containing 3 nmol of [^14^C]-C18:1-CoA (60 mCi/mmol), and in presence of 2 nmol of *lyso*-phospholipids. Reactions were initiated by addition of 100 μg of membrane proteins, and stopped by addition of 2 mL of chloroform/methanol (2:1, *v*/*v*) and 500 μL of water. Lipids were extracted as described below. The radioactivity incorporated into phospholipids was quantified using a Storm 860 (GE HealthCare).

### 4.5. Lipid Extraction

Polar and neutral lipids were extracted from bacteria or plants according to [55]. Phosphoinositide extraction from leaves was adapted from the method described by [56]. Briefly, 25 mg leaves were frozen into liquid N_2_ and ground using a tissue lyser. Aliquots of the frozen material were homogenated with 4 × 0.5 mL CHCl_3_/methanol/HCl 1M (1:2:0.1, *v*:*v*:*v*). Duplicate aliquots (725 μL) were mixed with 170 µL H2O. The samples were mixed thoroughly before adding 1 ng internal standard (1-stearoyl-2-arachidonoyl-PI(4,5)P2). Then, 725 µL CHCl_3_ and 170 µL HCl (2M) were added. After 5 min at room temperature, samples were centrifuged for 5 min at 5000 rpm and the lower organic phases were collected into fresh tubes. Then, 708 μL of prederivatization wash (CHCl_3_:MeOH: HCl (0.01M), 2:1:0.75, *v*/*v*/*v*) were added into samples before vortexing and centrifugation to separate the phases. The resultant lower phase were collected into fresh tubes before derivatization and LC-MS/MS analysis as described by [56].

### 4.6. Polar and Neutral Lipids Analysis

Neutral and polar lipids were separated by TLC using hexane/diethyl ether/acetic acid (90:15:2, by vol.) and chloroform/methanol/1-propanol/methyl acetate/ 0.25% KCl (10:4:10:10:3.6, by vol.) as the solvent respectively. Plates were then dipped for 5 min into 100 mL of PBS buffer added with 1mL of 0.1% (*w*/*v*) primuline in acetone:water (1:9, *v*:*v*), washed for 5 min in PBS buffer and dried for 5 min at 100 °C. Lipids were then visualized under UV light. The silica gel zones corresponding to the various lipids were then scraped from the plates and added to 1 mL of methanol/2.5% H_2_SO_4_ containing 5 *μ*g of heptadecanoic acid methyl ester. After heating at 80 °C for 1h, 1 mL of water was added and fatty acid methyl esters (FAMES) were extracted using 0.5 mL of hexane. Separation of FAMES was performed by GC (Agilent Technologie 7890A) on a 15 m × 0.53 mm Carbowax column (Alltech Associates, Deerfield, IL, USA) by flame-ionization detection. The oven temperature was programmed to be 160 ***°***C for 1 min, followed by a 20 ***°***C/min ramp to 190 ***°***C and a second ramp of 5 ***°***C/min to 210 ***°***C, and maintained at this temperature for a further 6 min. The retention times of FAMES were determined by comparison with standards, and they were quantified using heptadecanoic acid methyl ester as standard.

### 4.7. Histochemical GUS Assays

Whole seedlings or different tissue samples were analysed by histochemical assays for GUS activity as described somewhere else [57]. The plants were observed with a Leica binocular loupe (Leica MZ16F) coupled with a camera (DFC 420) and data processing was done with Leica application suite version 2.5.0 R1.

For histological analysis of seeds, dry or imbibed seeds were fixed in 4% paraformaldehyde, 4% DMSO in 10 mM PBS (pH 7), placed under vacuum for 15 min and incubated overnight at 4 °C before being dehydrated in alcohol and embedded in paraffin (Paraplast plus; Sigma-Aldrich). Eight µm-thick sections were prepared and were observed under a microscope (Zeiss-Axioplan).

### 4.8. Seed Coat Permeability

*Arabidopsis* dry seeds were incubated in the dark at 30 °C for 48 h in an aqueous solution of 1% (*w*/*v*) tetrazolium red (2,3,5-triphenyltetrazolium) [47]. Seeds were rinsed in water before imaging and percentage of positive seeds was calculated using image J software. Values represent the mean of coloured seeds (%) of 4 biological replicates from 3 independent experiments (*n* = 12)

### 4.9. Transient Expression System and Confocal Microscopy

Four-week-old tobacco (*N. tabacum* cv Petit Havana) greenhouse plants grown at 22–24 °C were used for *Agrobacterium tumefaciens* (strain GV3101)-mediated transient expression as described somewhere else [58]. Then, 48 h after infection of the lower epidermis, confocal analysis was performed on transformed leaves. Images were captured with the confocal laser scanning microscope Leica TCS SP2 with a ×63 oil immersion objective. For imaging of the coexpression of GFP and YFP constructs, we respectively used the Argon laser 488 nm and Argon laser 514 nm. To avoid communication and bleeding of fluorescence, both of the lasers were used alternatively with line switching on the multitrack facility of the microscope. Fluorescence emissions were detected at 495–525 nm for GFP and 535–590 nm for YFP.

### 4.10. Real-Time RT-PCR Conditions and Analysis

RNAs from leaves of wild-type (Col0), TDNA, overexpressing *LPIAT* line (LPIATOx) and rescue line (*lpiat*:LPIATOx) were extracted using the RNeasy plant mini kit (Qiagen). RNAs from seeds were extracted as described by [59]. Purified RNAs were treated with DNase I using the DNA- free kit (Ambion, now Invitrogen, http://www.invitrogen.com). RT PCR was carried out with SuperScript RT II kit (Invitrogen) using 1µg of total RNA as template and oligo(dT) as primers. To determine the relative transcript levels of *LPIAT*, RT-PCR assays were performed using the following oligonucleotides: GGGGTCAGCTTGAAGATTAAGA as a 5’-primer and CCAACATTGGCAGATTCAAC as a 3’-primer. All samples were assayed in triplicate wells and efficiency test was performed on primers. These analyses were done on two biological replicates. Real-time PCR was performed on an iCycler (Bio-Rad). Transcript abundance in samples was determined using a comparative Ct method, relative to Col0 samples. The relative abundance of ACT2 (At1g49240) and EIF4 (At3g13920) mRNA in each sample was determined and used to normalize for differences in total RNA amounts for expression level quantification in leaves. For seed samples, the normalization was realized using only EIF4 mRNA abundance. 

## Supplementary Materials

Supplementary materials can be found at https://www.mdpi.com/1422-0067/21/5/1654/s1. All authors have read and agreed to the published version of the manuscript.
