# Supplementary material for: LPIAT, a lyso-Phosphatidylinositol Acyltransferase, Modulates Seed Germination in Arabidopsis thaliana through PIP Signalling Pathways and is Involved in Hyperosmotic Response
Source: Int J Mol Sci. 2020 Feb 28;21(5):1654. doi: 10.3390/ijms21051654 (PMC7084726; doi:10.3390/ijms21051654)
Supplement: Supplementary file 1 [file ijms-21-01654-s001.zip › Figures supl revised4/Figure S1 - LPIAT gene structure.pdf]

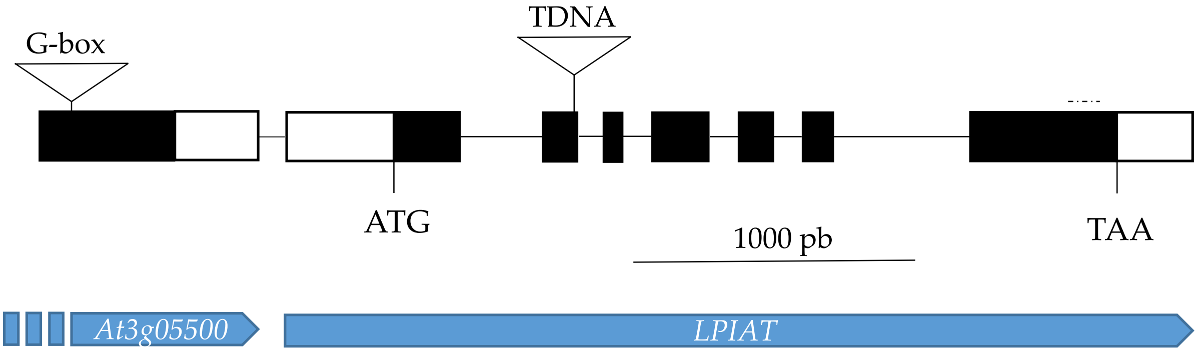


**Figure S1.** Structure of *LPIAT*. Black box, exons;  white box, untranslated regions; black line, introns. dashed line, sequence amplified for RT-qPCR experiment. T-DNA insertion is located within the exon 2., the position of a putative  G-box within the last exon of At3g05500g is showed.
