# Supplementary material for: LPIAT, a lyso-Phosphatidylinositol Acyltransferase, Modulates Seed Germination in Arabidopsis thaliana through PIP Signalling Pathways and is Involved in Hyperosmotic Response
Source: Int J Mol Sci. 2020 Feb 28;21(5):1654. doi: 10.3390/ijms21051654 (PMC7084726; doi:10.3390/ijms21051654)
Supplement: Supplementary file 1 [file ijms-21-01654-s001.zip › Figures supl revised4/Figure S2- cellular location.pdf]

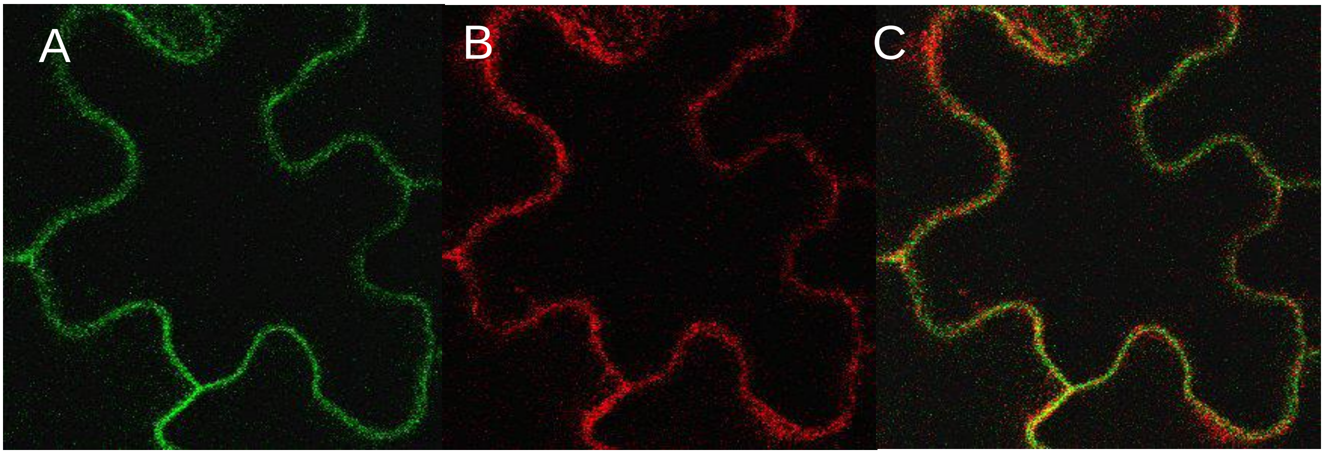

<u>Figure S2.</u> Localization of *LPIAT*–YFP in a tobacco leaf epidermal cell. (**A)** plasmalemma marker PMA4-GFP (green); (B): *LPIAT*-YFP (red); (**C)**: co-expression of *LPIAT*-YFP and PMA4-GFP in the plasmalemma of tobacco cell
