# Supplementary material for: LPIAT, a lyso-Phosphatidylinositol Acyltransferase, Modulates Seed Germination in Arabidopsis thaliana through PIP Signalling Pathways and is Involved in Hyperosmotic Response
Source: Int J Mol Sci. 2020 Feb 28;21(5):1654. doi: 10.3390/ijms21051654 (PMC7084726; doi:10.3390/ijms21051654)
Supplement: Supplementary file 1 [file ijms-21-01654-s001.zip › Figures supl revised4/Figure S3 - SDS PAGE gel.pdf]

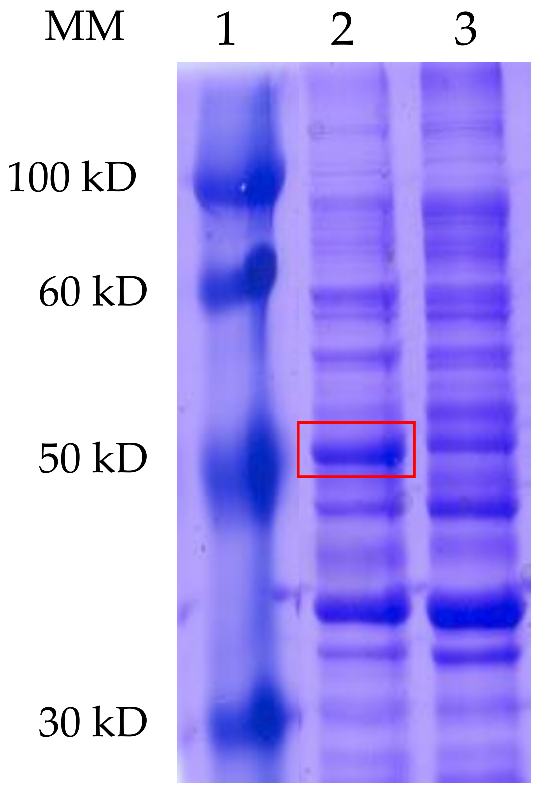

Figure S3. SDS-PAGE total protein analysis of microsomes from *E. coli* transformed by pET15b::*LPIAT* (lane 2) or empty pET15b (lane 3). MM, molecular marker (lane 1). 100 µL of microsomes were centrifuged and resuspended in 10 µL of loading buffer and loaded in a 12% polyacrylamide gel. After migration, proteins were revealed by Coomasie blue.
