# Supplementary material for: LPIAT, a lyso-Phosphatidylinositol Acyltransferase, Modulates Seed Germination in Arabidopsis thaliana through PIP Signalling Pathways and is Involved in Hyperosmotic Response
Source: Int J Mol Sci. 2020 Feb 28;21(5):1654. doi: 10.3390/ijms21051654 (PMC7084726; doi:10.3390/ijms21051654)
Supplement: Supplementary file 1 [file ijms-21-01654-s001.zip › Figures supl revised4/Figure S4- qPCR seeds and leaves.pdf]

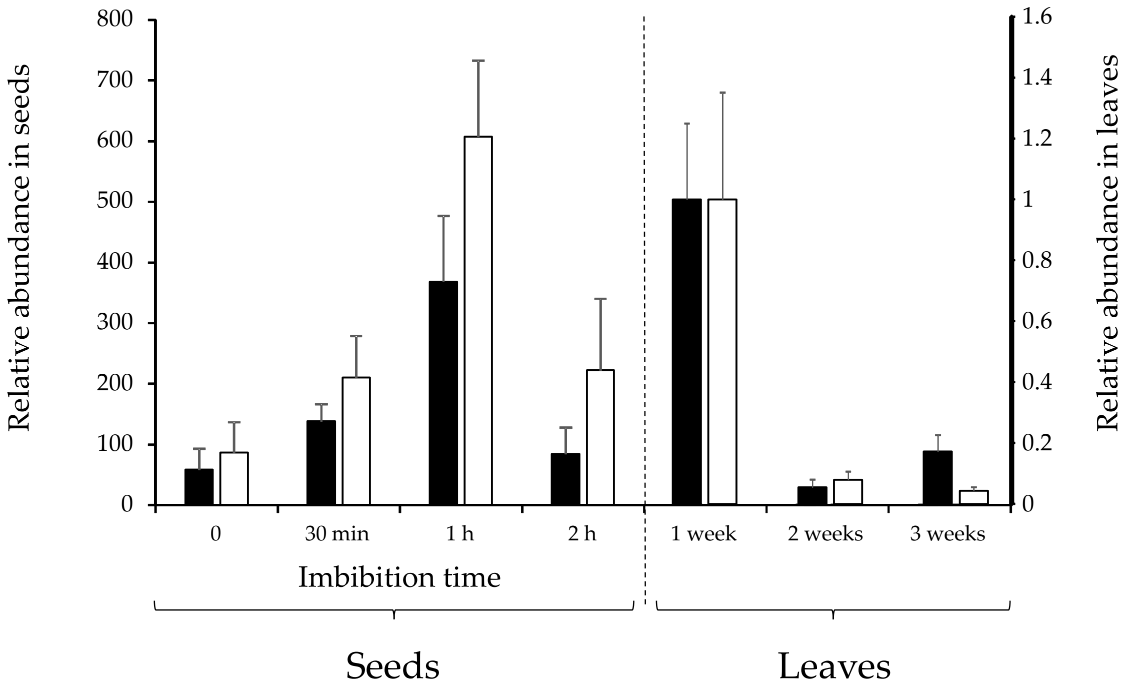

Figure S4. Determination of *LPIAT* mRNA levels by real-time PCR in seeds upon imbibition and in leaves in wild-type. Values represent mean ± SD of three technical replicates for two biological replicates (black and white boxes). Relative expression quantities are represented related to one-week old leaves level, which was set to one.
