# Supplementary material for: LPIAT, a lyso-Phosphatidylinositol Acyltransferase, Modulates Seed Germination in Arabidopsis thaliana through PIP Signalling Pathways and is Involved in Hyperosmotic Response
Source: Int J Mol Sci. 2020 Feb 28;21(5):1654. doi: 10.3390/ijms21051654 (PMC7084726; doi:10.3390/ijms21051654)
Supplement: Supplementary file 1 [file ijms-21-01654-s001.zip › Figures supl revised4/Figure S5- Lipid Phenotype seed.pdf]

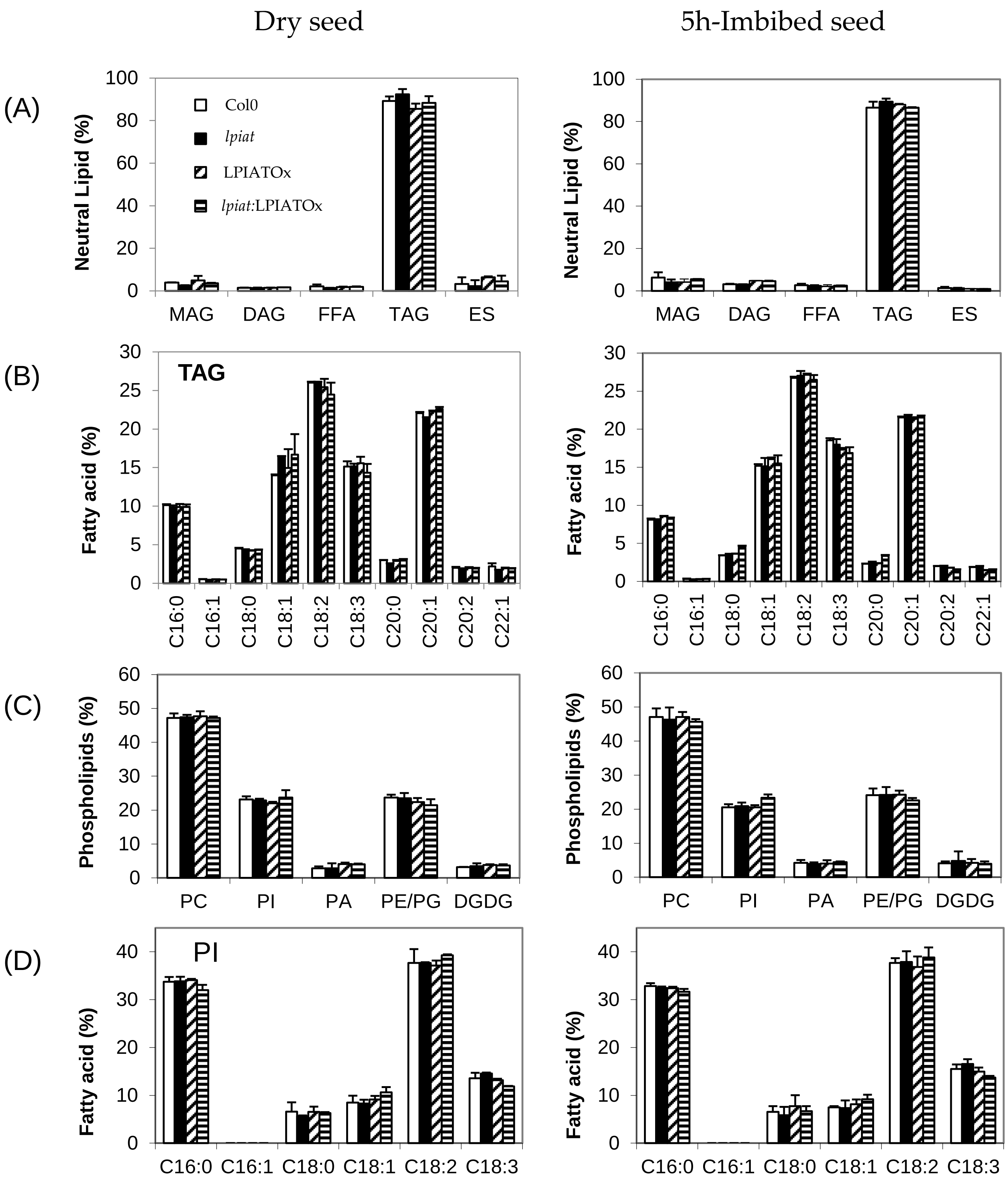

Figure S5. Lipid profile of *LPIAT* mutant seeds. 50 seeds and 25 mg seeds were used for neutral lipid and polar lipid quantification, respectively. Lipids were extracted from dry seeds or after 5h imbibition, separated by TLC, and quantified by GC-FID after transesterification. Values are mean ± SD (n=6)
(A) Neutral lipid composition; (B) Fatty acid composition of TAG; (C) Polar lipid composition; (D) Fatty acid composition of PI
