# Supplementary material for: LPIAT, a lyso-Phosphatidylinositol Acyltransferase, Modulates Seed Germination in Arabidopsis thaliana through PIP Signalling Pathways and is Involved in Hyperosmotic Response
Source: Int J Mol Sci. 2020 Feb 28;21(5):1654. doi: 10.3390/ijms21051654 (PMC7084726; doi:10.3390/ijms21051654)
Supplement: Supplementary file 1 [file ijms-21-01654-s001.zip › Figures supl revised4/Figure S6 LPIAT expression in seed.pdf]

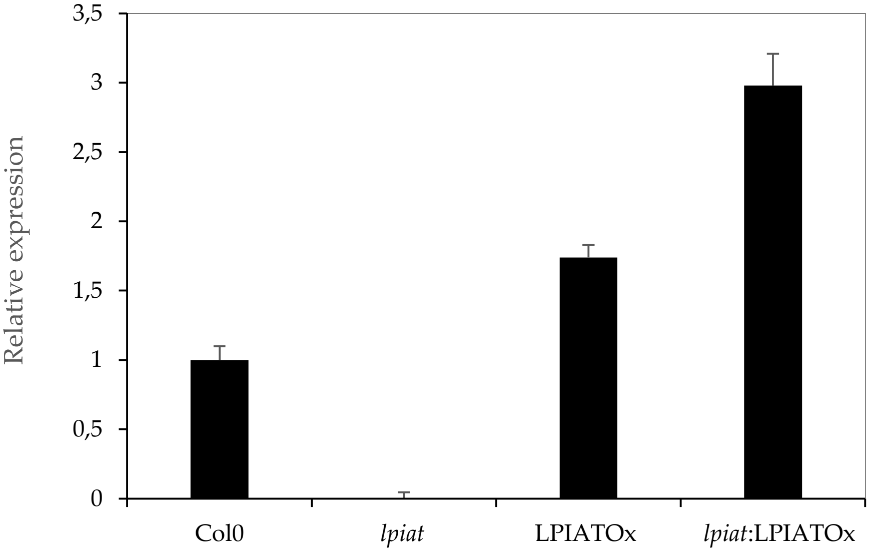

Figure S6. Expression of *LPIAT* in seed from wild-type, *lpiat*, overexpressing (LPIATOx) and rescue (*lpia*t:LPIATOx) lines. Values represent mean ± SD of three technical replicates, representative of two biological replicates. Relative expression quantities are represented related to wild type level, which was set to one.
