# Supplementary material for: LPIAT, a lyso-Phosphatidylinositol Acyltransferase, Modulates Seed Germination in Arabidopsis thaliana through PIP Signalling Pathways and is Involved in Hyperosmotic Response
Source: Int J Mol Sci. 2020 Feb 28;21(5):1654. doi: 10.3390/ijms21051654 (PMC7084726; doi:10.3390/ijms21051654)
Supplement: Supplementary file 1 [file ijms-21-01654-s001.zip › Figures supl revised4/figure S7 - tetrazolium.pdf]

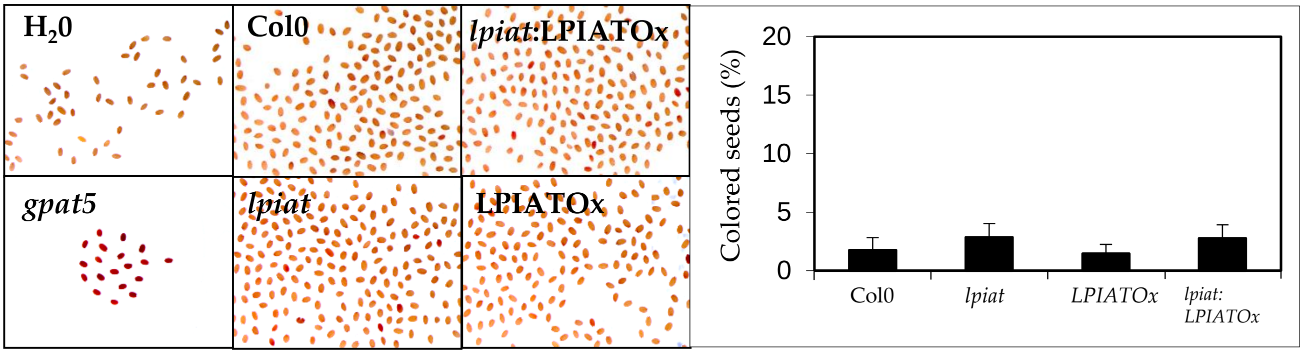

Figure S7. Permeability of *LPIAT* mutant seeds is not altered. Arabidopsis dry seeds were incubated in the dark at 30°C for 48 h in an aqueous solution of 1% (w/v) tetrazolium salt. *gpat5* mutant was used as positive control. Percentage of positive seeds was calculated using image J software. Values are means ± SD of 4 biological replicates from 3 independent experiments
